# Actinomycetes from Caves: An Overview of Their Diversity, Biotechnological Properties, and Insights for Their Use in Soil Environments

**DOI:** 10.3390/microorganisms10020453

**Published:** 2022-02-16

**Authors:** Beatrice Farda, Rihab Djebaili, Ilaria Vaccarelli, Maddalena Del Gallo, Marika Pellegrini

**Affiliations:** Department of Life, Health and Environmental Sciences, University of L’Aquila, Via Vetoio, Coppito, 67100 L’Aquila, Italy; beatrice.farda@guest.univaq.it (B.F.); ilaria.vaccarelli@student.univaq.it (I.V.); maddalena.delgallo@univaq.it (M.D.G.)

**Keywords:** microbiota, culturable actinomycetes, bioactive compounds, antimicrobial activity, salt stress, alkaline stress, mineral solubilisation, bioremediation, biostimulant

## Abstract

The environmental conditions of caves shape microbiota. Within caves’ microbial communities, actinomycetes are among the most abundant bacteria. Cave actinomycetes have gained increasing attention during the last decades due to novel bioactive compounds with antibacterial, antioxidant and anticancer activities. However, their potential role in soil environments is still unknown. This review summarises the literature dealing with actinomycetes from caves, underlining for the first time their potential roles in soil environments. We provide an overview of their diversity and biotechnological properties, underling their potential role in soil environments applications. The contribution of caves’ actinomycetes in soil fertility and bioremediation and crops biostimulation and biocontrol are discussed. The survey on the literature show that several actinomycetes genera are present in cave ecosystems, mainly *Streptomyces*, *Micromonospora*, and *Nocardiopsis*. Among caves’ actinomycetes, *Streptomyces* is the most studied genus due to its ubiquity, survival capabilities, and metabolic versatility. Despite actinomycetes’ outstanding capabilities and versatility, we still have inadequate information regarding cave actinomycetes distribution, population dynamics, biogeochemical processes, and metabolisms. Research on cave actinomycetes needs to be encouraged, especially concerning environmental soil applications to improve soil fertility and health and to antagonise phytopathogens.

## 1. Introduction

**Figure 1:**
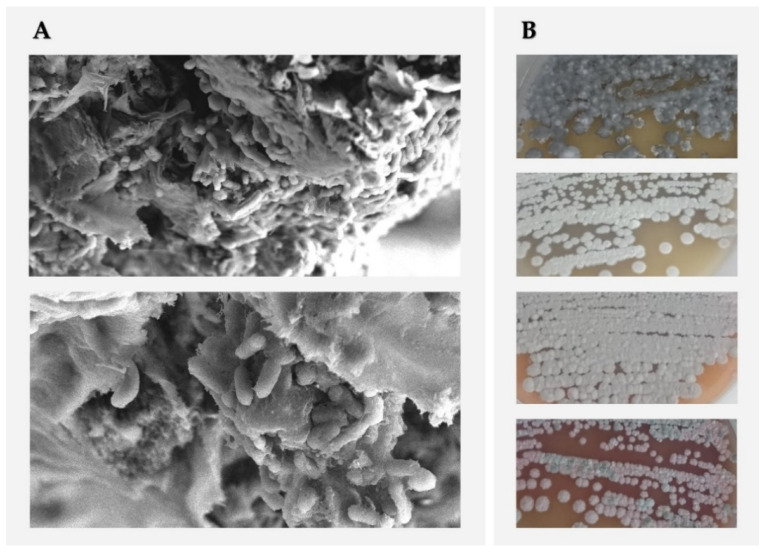
Actinomycetes’ biofilm and aggregations’ structures formed on surfaces visualised by scanning electron microscopy (**A**) and growth shapes and colours of spores formed on agar plates of international streptomyces project n°2 (**B**).

Actinomycetes are prokaryotic organisms regrouped into the Bacteria taxonomic group, playing an essential role within the microbiota of all environments [1]. This contribution is significant under unfavourable conditions, such as saline and alkaline habitats, drought stress, and high temperatures. The cellular characteristics (i.e., Gram-positive, elongated cells forming filamentous or hyphal structures, and spores’ formation) and metabolic versatility allow these bacteria to be present and survive in a wide range of soil environments [2]. Figure 1 shows the diverse growth characteristics shown by some actinomycetes and their ability to form biofilm structures and aggregations.

Studies concerning the use of bacteria, individually or in a consortium, may concern the use of autochthonous or allochthonous organisms to improve the soil environment status [3]. Depending on the purpose of the application, we can isolate microorganisms from the final application site or another site with peculiar characteristics. For actinomycetes used for agricultural purposes, the source of isolation can be the rhizosphere of plants grown at the re-application site to improve their subsequent reuse [4]. However, if our goal is to isolate bacteria with specific characteristics, we must look elsewhere to where the probability of finding them is much higher. The latter is the case of phosphate solubilising bacteria.

Phosphate solubilising bacteria isolation is more probable from alkaline environments (pH > 8), often inhospitable for cultivation because of nutrient imbalances. However, these bacteria have a wide range of applications in different agricultural soils to provide the availability of this essential element for plant absorption. These situations lead most of the time to the search for and application of allochthonous bacteria. Among the environments that induce the selection of actinomycetes with unique characteristics, there are the hypogean environments, particularly caves.

Caves are rich in carbonates, sulphates, phosphates, and potassium-rich sediments, with diverse but approximately stable temperatures and humidity [5,6]. These conditions shape a unique mineral solubilising microflora. Soil mineral solubilising microflora transforms complex and insoluble forms of minerals into simple nutrients [7]. This capability is significant in biogeochemical cycling and is a sustainable approach to improving crop yields. Mining soil remediation is promoted by mineral solubilising microflora, which speeds up mineral breakdown and soil restoration [8]. The adaptation of Actinobacteria to extreme environments and the associated interactions have led to the evolution of different biosynthetic potentials. Under harsh growth conditions, actinobacteria produce biosurfactants, which accelerate biological oxidation and pollutant biodegradation. Bacteria with these resistance mechanisms produce superoxide dismutase, efflux transporters, and metal-binding proteins. These resistance mechanisms allow bacteria to degrade herbicides, pesticides, heavy metals, petrochemicals, and some aromatic compounds [9]. Microbial-induced and biological-mediated calcification mechanisms mediate CO_2_ sequestration, producing natural carbon sinks important in non-calcareous soils [10].

The caves’ environmental conditions also shape microbial secondary metabolites’ production. Several authors have reviewed actinomycetes, mainly antibiotics, from caves for their bioactive compounds. Actinomycetes are the most important producers of antibiotics and a range of anticancer, anthelminthic, antifungal, and immunosuppressive drugs [11,12,13,14]. Actinobacteria produce enzyme inhibitors with various industrial and biotechnological applications [15]. Moreover, they are involved in nanoparticle synthesis with biopotential activities [16]. The recent review of Rangseekaew and Pathom-aree underlined that cave environments harbour novel and diverse actinobacteria (mainly *Streptomyces* spp. [17] with novel bioactive compounds with a broad spectrum of activities [18]. Within this context, we hypothesised that actinomycetes selected from these environments could be an essential source for soil environments in shaping and inducing enhancements in fertility, and the health of soils. The purpose of this review is to underline the potential roles of actinomycetes in soil environments and stimulate research to advance knowledge on the topic. To evaluate the suitability of caves’ actinomycetes to be used in soil environments, we summarised the literature dealing with actinomycetes from caves. Their diversity and their role in antagonism against phytopathogens, soil fertility improvement, remediation of contaminated soils, and improvement of crop productivity are described. The relevant publications on actinomycetes were searched using several databases, with a total of 121 relevant articles being included in the final electronic library. This review provides, for the first time, useful information for future research on caves’ actinomycetes as soil environment improvement agents.

## 2. The Diversity of Actinomycetes from Caves

Actinomycetes exhibit considerable diversity in caves because of the environmental stresses, which shape microbiota and pave the development of new species. Cave ecosystems drive unique evolutionary stressors, and the scarcity of energy determines complex interactions between different microorganisms. For these reasons, caves are fascinating and promising places, especially for investigating novel actinomycetes [19]. Most of the actinomycetes can be seen by the naked eye adhering to the rock surface of the ceiling and wall rocks with colonies from 1 to 10 mm in diameter [20,21,22]. The work of Long and collaborators showed the differences among culturable and non-culturable actinobacteria within Shuanghe Cave (Asia); even if culture-dependent methods led to unrepresentative results of microbial communities, their study provided supplemental results and allowed to obtain several antimicrobial strains useful in biotechnology [23]. As observed by Pašić and collaborators [20], sometimes the composition of cave wall microbial communities can share similarities to microbial formations appearing within human-impacted caves. This aspect might be a notable facet to evaluate when investigating secondary metabolite production of actinomycetes, assuming these bacteria are often observed in cave parietal biofilms. Is there a noteworthy difference in bioactive compounds’ production between pristine caves or human-impacted caves? Even if we believe we must study cave microbiology comprehensively, focusing on this aspect could help us define more suitable environments and/or characteristics shaping biota of more relevant interest for biotechnological purposes. Indeed, various authors attest to the importance of pristine caves for searching novel microbial species and bioactive compounds [19]. This aspect can also have implications in conserving impacted cave environments, aiming to preserve what shapes the invisible resource. Ultimately, the advent of NGS techniques could represent a powerful tool in determining actinomycetes’ diversity and beyond.

**Figure 2:**
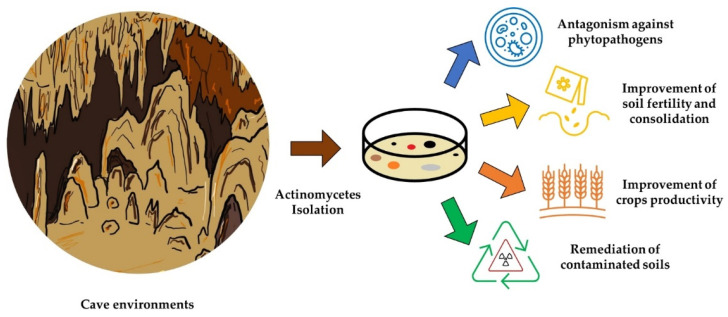
Schematic summary of the possible uses of actinomycetes isolated from cave environments.

Until the last decades, much of the identification of the actinomycetes species has been based on chemical and cultural techniques, conceding a large portion of diversity behind them. With the advent of new sequencing techniques, it will be possible to obtain more information about the subterranean microbial diversity and meld the information deriving from cultivation techniques to succeed in cultivating non-cultivable cave microorganisms. These activities can thus constitute the basis for comprehensive knowledge of cave actinomycetes and an industrialisation perspective of the strains of interest for different and disparate biotechnological applications. Figure 2 summarises the possible uses of actinomycetes isolated from cave environments.

Many studies have focused investigations on actinomycetes’ isolation from cave-culturable communities, discovering novel species with interesting biological properties. Table 1 summarises the diversity of actinomycetes obtained from these studies.

The *Streptomyces* genus mainly constitutes the culturable microflora. Beyond *Streptomyces*, the other most common genera are *Micromonospora* and *Nocardiopsis*, and the isolation of rare genera (e.g., *Kocuria*) is frequent. According to the biological trait investigated, many authors found effective strains belonging to other genera different than *Streptomyces*. Based on the intrinsic and extrinsic characteristics of the samples, isolation is performed differently. The isolation media needs to be designed and selected considering the key characteristics of the sample and the aim of the isolation, selecting inhibitors for unwanted microflora, and addressing the isolation towards special actinomycetes [2]. For example, if we aim to isolate thermotolerant actinomycetes, the sample must be air-dried at room temperature (7–10 days) and then subjected to a heat treatment of 120 °C for 1 h. The optimisation of the isolation medium is also important. For instance, if we want to isolate halotolerant/halophilic actinomycetes, the medium salt concentration must be around 15–25%. If our goal is to isolate alkalitolerant/alkalophilic actinomycetes, the medium selected needs to be adjusted to a pH of 10–12. The medium optimisation is also performed to select peculiar species among the isolates (e.g., the addition of heavy metals to select heavy metal-resistant strains).

Studies aimed at isolating actinomycetes from caves are mainly related to their bioactive compounds’ production, especially antibiotics. Among these studies, the manuscript of Long and collaborators isolated a total of 239 actinomycetes from Shuanghe Cave (Asia) [23]. Based on morphological characteristics of hyphae and spores, only 23 isolates were subjected to DNA barcoding by 16S rRNA gene sequencing. The main genus found was *Streptomyces*, accounting for 52% of the 23 isolates, followed by *Actinoplanes* with an abundance of 13%. Among the 23 isolates, the authors also reported two putative novel species. These new species were closely related to *Streptomyces* and *Micromonospora* genera. The other strains belonged to the genera *Nocardioides* (8.7%), *Micromonospora* (8.7%), *Agromyces* (8.7%), *Oerskovia* (4.4%), and *Rhodococcus* (4.4%). Other work has aimed to isolate actinomycetes from caves as well as for heavy metal stress, salinity tolerance, and mineralisation capabilities. Hamedi and collaborators isolated 76 actinomycetes from 22 of the 33 samples collected from different sections of the Hampoeil cave (Iran) [24]. The 16S rRNA gene barcoding showed that over half of the isolates belonged to the *Streptomyces* genus (54%), followed by *Micromonospora* (18.4%). The other isolates belonged to *Micrococcus* (5%), *Kocuria* (4%), and *Corynebacterium* (5%). Groth and collaborators isolated several strains from soils, walls, and stalactites of Grotta dei Cervi (Porto Badisco, Italy). Even if many of them were unidentified, the isolates belonged mainly to the *Streptomyces* genus (45%, in soils, 75% in walls, and 8% in stalactites). The other genera found were *Brevibacterium*, *Amycolatopsis*, *Nocardia*, *Gordonia* were, and *Nocardiopsis* [25,26].

The complete description of microbial communities can be achieved by next-generation sequencing. Riquelme and collaborators carried out one of the most complete and extended works describing the microbiota of caves [22]. The authors studied the diversity of volcanic cave actinomycetes in Spain, Portugal, USA, and Canada. The same approach was carried out by many other authors of different cave systems in various parts of the world. Table 2 summarises the most abundant actinomycetes found in caves by these studies and their relative abundances in microbial communities

From this survey, it was possible to underline that actinomycetes account for 2–93% of cave bacterial communities, based on the sample investigated. The most abundant genus is *Streptomyces*, followed by *Micromonospora*, *Microbacterium*, *Micrococcus*, *Nocardioides*, *Agromyces*, *Rhodococcus*, and *Saccharothrix.* The different genera account for different percentages of the actinomycetes community based on the type of cave and environmental stressors present.

## 3. Expansion of Productive Landscapes

**Figure 3:**
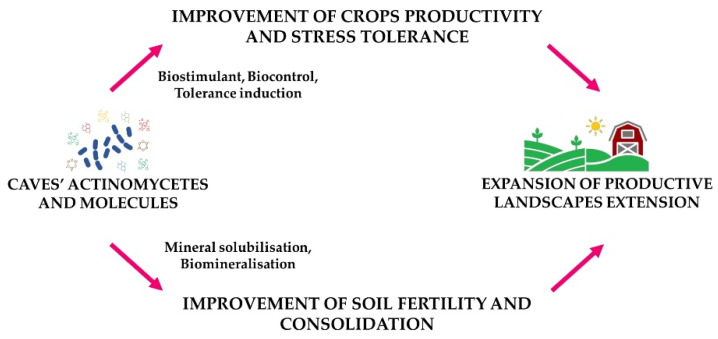
Schematic summary of the caves’ productive landscape expansion.

Climate change effects and anthropogenic activities associated with urbanisation and non-food crop cultivation are expected to limit the landscapes dedicated for food production. The major threats are represented by the spread of phytopathogens, excessive use of agrochemicals, and decreases in soil fertility. As presented in Figure 3, caves’ actinomycetes could be a useful tool to overcome these problems and extend productive landscapes by improving the productivity and stress tolerance of crops and improving soil fertility and consolidation. These activities are described in detail in the following sections.

### 3.1. Antagonism against Phytopathogens

With the emergence of multi-resistant pathogenic microorganisms, the search for new antimicrobial metabolites has become one of the primary goals of sustainable agriculture. To increase the possibility of discovering novel metabolites, research must shift towards extreme and under-exploited ecosystems, such as caves. The production of secondary metabolites is related to the phenomenon of *quorum sensing* promoted by biofilms, an indispensable ecological response to survive and cope with the limiting environmental pressures of the caves [37]. Cave actinomycetes are an excellent resource of new bioactive compounds with several and complex structures [10,38]. Actinobacteria produce about two-thirds of known antibiotics, most of them belonging to the *Streptomyces* genus [1]. Several actinobacteria are responsible for a wide range of bioactive compound productions, such as enzyme inhibitors, immune modifiers, plant growth-promoting substances, and natural dyes, and they exert several antimicrobial, antifungal, anticancer, antiparasitic, and immunosuppressant activities [1,18,39,40,41,42]. So far, studies and reviews have been mainly based on applying these compounds against human pathogens [18,43]. These studies report a broad spectrum of effectiveness against Gram-positive (e.g., *Bacillus subtilis* and *Staphylococcus aureus*) and Gram-negative bacteria (e.g., *Pseudomonas aerungiosa*), and fungi (e.g., *Candida albicans*) [30]. Several studies are needed to show that this antimicrobial activity is also effective against phytopathogens. However, the results achieved so far are promising. The recent study of Axenov and collaborators described the effective use of metabolites extracted from *Streptomyces* spp., isolated from a Siberian conglomeratic karstic cave, against *Fusarium verticilloides* DSM 62264, the *Zea mays* rotting stalk’s causal agent associated with human diseases [44]. Culturable bacteria (*Bacillus*, *Nissabacter*, *Dickeya*, and *Serratia*) isolated from seven caves of Brazil showed effective in vitro inhibition against phytopathogenic strains of *Xanthomonas citri* subsp. *citri*, *Fusarium oxysporum*, and *Colletotrichum lindemuthianum* [45].

The investigations on the actinomycetes’ communities in Four Windows Cave revealed a diverse community of bacteria, including actinomycetes, producing secondary repellent compounds to invertebrates [46] and thus were helpful in counteracting pathogenic invertebrates. Molecular studies identified multi-domain enzymes responsible for natural product production with antimicrobial activities. These studies investigated the presence and diversity of antimicrobial biosynthetic polyketide synthase (*PKS*) and non-ribosomal peptide synthetase (*NRPS*) genes, and their clusters within the cave microbiome [47]. The presence of these enzymes was detected in the caves’ microbiome of Belgium [48], India [11,49], Bahamas [50], and Georgia [51]. Beyond antimicrobial compounds, cave actinomycetes present many other assets to counteract phytopathogens, i.e., siderophores and other enzymes [52,53]. These characteristics are effective against microbial phytopathogens [54] and have led to the speculation on the use of cave actinomycetes as biocontrol agents against phytopathogens.

### 3.2. Productivity of Crops Improvement

The scientific literature is lacking studies on the use of cave actinomycetes to improve crop productivity. However, there are many reports of actinomycetes isolated from other environments acting as effective plant biostimulants. These reports, extensively summarised by many authors [55,56,57], describe the isolation of actinobacteria, also from extreme environments, and their applications under stressful conditions (e.g., heavy metal stress and desertic, salty, and alkaline soil environments). Actinomycetes with a positive effect on plant growth and crop productivity belong to the plant growth-promoting actinobacteria (PGPA), a subgroup of the larger group of plant growth-promoting bacteria (PGPB). Regardless of the type and characteristics of the association, the mechanisms of action by which beneficial soil microorganisms can promote and/or maintain the physiological and phytosanitary status of the plant fall into two broad categories: direct and indirect mechanisms [58].

Direct mechanisms include atmospheric nitrogen fixation, plant hormone production, and the solubilisation of essential elements in plants. Atmospheric nitrogen fixation is a process carried out by microorganisms, both freely in the soil and in association or symbiosis with plants, that enables the reduction of atmospheric nitrogen and makes it available for microbial biosynthesis and plants. The production of plant hormones is promoted by the association of plants and microorganisms, leading to changes in the hormonal homeostasis of plants. Numerous microorganisms can synthesise or metabolise phytohormones, such as auxins, ethylene, cytokinins, gibberellins, abscisic acid, jasmonic acid, and salicylic acid, or influence hormone synthesis in plants [59]. The growth, development, and maintenance of the cellular processes of plants are ensured not only by nitrogen and hormones but also by essential elements such as phosphorus and potassium. These elements are hardly present in the soil and can hardly be taken up directly by the plants, as they are usually in inaccessible forms. As already described in the previous section, the availability of the accessible forms is ensured by the direct action of beneficial microorganisms, which, by directly dissolving the insoluble forms blocked in the minerals, promote the release of forms that can be taken up by the plants [7].

The indirect promotion of plant growth occurs when PGPB reduces or prevents a phytopathogenic organism’s harmful effects. Mechanisms of indirect promotion include synthesis of antibiotics and other antagonistic molecules that counteract the growth and development of the pathogen, secretion of siderophores, production of lytic enzymes, and competition for nutrients, as well as space in niches. Siderophores are small molecules with a high affinity for iron, forming complexes with this metal that make it unusable for microorganisms not part of the plant’s ecosystem. The development and spread of some phytopathogenic pathogens are closely linked to the presence of iron. Therefore, the action of siderophores produced by beneficial microorganisms indirectly prevents the development of pathogens by preventing them from developing, as they cannot use the bound iron [60].

The production of lytic enzymes has an antagonistic effect against some significant components of the cell walls of pathogenic organisms (e.g., the activity of the enzyme chitinase on the chitin of pathogenic fungi) [58]. By competing for nutrients, PGPBs counteract the development of pathogens in the environment of plants. By competing for ecological niches, the beneficial microorganisms prevent pathogenic bacteria or fungi from entering the niche, limiting their growth and development and thus their pathogenic activity [60]. In addition to the direct and indirect mechanisms, beneficial microorganisms can help plants improve their response to biotic stresses such as pathogens, but also abiotic stresses such as salt, water, or temperature stress through induced systemic resistance (ISR) [61]. The resistance mechanisms acquired by plants can be limited to the damaged organ or spread systemically throughout the plant. The latter include systemic acquired resistance (SAR) and ISR. B pathogens and parasites trigger SAR, while beneficial microorganisms mediate ISR in the rhizosphere [62]. Mineral dissolution abilities, production of bioactive molecules, competitive and antagonistic behaviour, and involvement in nutrient cycling have shown that cave actinomycetes are a promising tool for biological stimulation of plants, providing nutrition and the protection of crops.

### 3.3. Improvement of Soil Fertility and Consolidation

Soil fertility is threatened by diverse biotic and abiotic stresses. Salinisation and alkalinisation negatively affect agricultural productivity, microbial communities, and agricultural production among abiotic stresses.

Soil salinisation, induced by the accumulation of water-soluble salts, is a problem common to all zones of the Earth with different pedoclimatic conditions [63]. Halotolerant/halophilic microflora can survive in salty soils due to several mechanisms [64]:

- Specific composition of cellular membranes or cell walls that obstruct the input of high salt concentrations.
- Pumping ions out of the cell by a Na^+^/H^+^ anti-porter or use of K^+^/Na^+^ ion transporters for regulation of intracellular ionic concentration and osmotic adaptation.
- Endogenous compatible solutes biosynthesis and accumulation (e.g., sucrose, glycine betaine, and glycosyl glycerol).
- Adaptation of proteins and enzymes that produce high contents of solute ions.
- Enhancement of cell energy.
- Production of exopolysaccharides that helps the development of biofilms and blocks the entry of high salt into the cell.

Halotolerant/halophilic actinomycetes are usually found in extreme environments with high salt concentrations (e.g., seawater, saline soils, salt lakes) [65,66]. Anchialine caves present steep salinity gradients and can be a good source of novel halophilic/halotolerant actinomycetes. Hodges and collaborators isolated several actinomycetes from Bahamas anchialine cave systems [50]. Among the eleven isolates, four strains were closely related to the *Solwaraspora* species, while others were related to *Nocardiopsis*, *Micromonospora*, *Streptomyces*, and *Pseudonocardia*. These halophilic/halotolerant microorganisms can counteract the salinity’s adverse effects, restore saline degraded soils, and induce halotolerance in plants [67]. One of our recent studies highlighted the use of saline soils, *Streptomyces* and *Nocardiopsis* strains, as salt-tolerance inducers in *Triticum durum* under different salt concentrations (i.e., 0, 0.25, 0.5, 0.75, 1, 1.25, and 1.5 M NaCl) [68]. Microorganisms induce salinity tolerance through stress-responsive genes stimulation, mineral solubilisation, volatile and antioxidant compounds’ production, phytohormones’ regulation, and regulation of turgor pressure, homeostasis of ions, and osmotic balance (e.g., proline) [69].

Soil alkalinisation is caused by high concentrations of carbonates (CO_3_^2−^) and bicarbonates (HCO^3−^), leading to desertification in many soils [70]. Alkalitolerant/alkaliphilic bacteria can survive in extracellular environments with a pH up to 11 and mitigate alkalinity stresses in soil environments [71]. Fang and collaborators revealed that alkaline pH promoted the isolation of *Streptomyces* spp. and rare actinomycetes’ strains from karstic caves [31]. They found that *Actinobacteria* growth was implemented with the introduction of different calcium salts as CaCO_3_, CaCl_2_, and (CH_3_COO)_2_Ca at 0.01%, 0.1%, and 1% (w/v). Application effectiveness of alkalitolerant/alkaliphilic actinomycetes in soil environments remains uninvestigated. However, it is well known that these microorganisms:

- Increase availability of assimilable iron [72];
- Release nutrients from minerals [73];
- Decompose recalcitrant biopolymers [74];
- Complete degradation of nitriles [75];
- Enhance rock weathering [76];
- Recycle humic acids [77];
- Degrade hydrocarbons [41].

Based on these abilities, alkalitolerant/alkaliphilic actinomycetes could play an essential role in improving soil fertility, expanding productive landscape extension. Mineral solubilisation covers a relevant role in salty and alkaline soils. As already described, this capability is essential in soil environments’ biogeochemical cycling and allows for the conversion of nutrients blocked in mineral forms into available forms [7]. Cave solubilising actinomycetes could be used as bioinoculants to improve soil nutrient availability and convert inhospitable soils into agricultural soils. The in silico investigation on the microbial communities of five Indian caves, mainly composed of proteobacteria and actinobacteria, revealed genes involved in carbon, nitrogen, and methane metabolisms, and complex metabolic pathways for the bacterial community survival in nutrient-limited cave environments [34]. Actinomycetes also participate to organic materials’ cycling through the production of hydrolytic compounds [78]. This activity is relevant for the breaking down of chitin, cellulose, and lignin found in soil environments. The breakdown of these compounds releases nutrients exploitable by other organisms. Other organisms also carry out this activity. However, actinomycetes present some advantages over other microorganisms. The metabolic versatility, cell structure, and sporulation ability allow actinomycetes to survive under adverse conditions [79]. The use of thermotolerant actinomycetes-base inoculants during different stages of composting has already been demonstrated to be effective in cellulose degradation and the increase in humic matter content [80]. These aspects highlight the usefulness of cave actinomycetes in improving biogeochemical cycling.

Another potential effect on soil is linked to the biomineralisation ability of actinomycetes. Beyond the described production of natural carbon sinks that help CO_2_ sequestration [10], biomineralisation could help consolidate soil structure. The relatively stable abiotic conditions defining caves allow a privileged investigation of the relationships between the geochemical processes and the microbial communities’ activity. By interacting with the minerals in caves, specific microbial communities play an essential role in forming caves, actively shaping the concretions, or participating passively, stratifying dead cells within calcite layers. Microorganisms can trigger different reactions with the rock matrix, determining minerals’ formation and/or dissolution through many biochemical reactions and redox transformations [81]. Moonmilk is a typical white secondary deposit, generally observed within caves in limestone. It is mainly composed of carbonate minerals, comprising different morphologies with crystals or filaments that could show lengths from micrometres to nanometres. These peculiar formations can cover diverse substrates in caves, such as walls and ceilings. Depending on their water content, they can also show different textures, from muddy to drier consistencies [81]. Although the origin of moonmilk has been considered for several years to be controversial, it is commonly accepted that the active microbiological features contribute to the formation of these secondary depositions. This theory was reinforced by several microbial investigations related to these secondary deposits [48]. Different studies have shown that the moonmilk hosts a rich microbiota, mainly composed of bacteria, fungi, and archaea. Borsato and collaborators also analysed changes in the moonmilk textures. They observed a correspondence on the state of microbial activity: wet traits are generally related to an active community, and the moonmilk tend to become drier when microbial activity is less active [82]. Therefore, investigating microbial communities in different moonmilk textures could provide further information on the microorganisms involved in this specific biomineralisation process. Cañaveras and collaborators argued the evidence of a crucial role of actinobacteria in the moonmilk formation [83]. These bacteria play a significant role in biofilm formation and promote calcium carbonate precipitation by creating locally favourable conditions and using their cell walls as nucleation sites. This theory has been corroborated also by Bindschedler and collaborators [84] as well as Li and collaborators [85]. Park and collaborators found *Streptomyces* solely in dry moonmilk specimens within Baeg-nyong Cave (South Korea) [86]. The authors discussed that the secondary metabolites produced by Actinomycetes might directly inhibit or promote the growth of other microorganisms [87].

Actinomycetes’ secondary metabolites may also act as cell-signalling molecules, with an essential role in microbial community maintenance [88]. Through their powerful properties, calcifying bacteria have been used from the 1990s to develop different biotechnological applications, especially in the bioconstruction and biorestoration fields. Specifically, bioconsolidation prevents and/or stabilises erosion and increases slope stability of different soils. This method relies on biochemical processes naturally occurring in environments to improve the engineering properties of soils [89]. However, various solutions to these problems in the geotechnical field do not consider biotechnological solutions. The conventional methods mainly rely upon the application in the soil matrix of cement or chemicals. Even if effective, these techniques can permanently pollute soils and water environments and be more expensive than bioconsolidation solutions [90]. Even if the applicability of biological treatments is still minimal, a possible answer to the whole criticalities is represented by calcifying bacteria [91]. For instance, Whiffin and collaborators tested the capabilities in the reinforcements of sands by the treatment with different calcifying bacteria and showed a decreased porosity and improved soil strength in the biotreated soils [92]. Instead, Ivanov and Chu compared the cost of conventional chemical groutings with microbial-related techniques and found the latter to notably cheaper than the conventional ones [93].

These findings show that calcifying bacteria in the bioconsolidation of soil is a promising and fascinating eco-friendly alternative approach capable of solving environmental problems in multidisciplinary fields. Specifically, actinomycetes isolated from cave environments that show biomineralisation capabilities could represent a valuable resource for several fields, enhancing the actual properties that industrialised stains detain. A captivating aspect is clearly linked, for instance, to the genus, *Streptomyces*, found in dry moonmilk formations, which could represent a valuable role in the bioconsolidation of soils with low water content.

## 4. Remediation of Contaminated Soils

**Figure 4:**
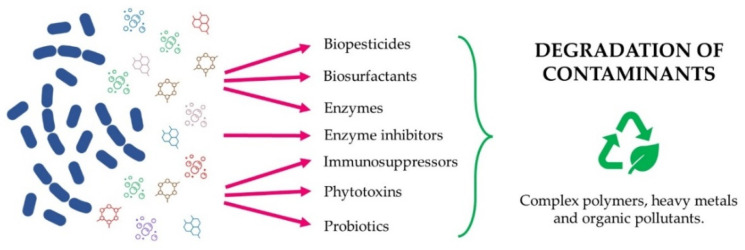
Schematic summary of the uses of actinomycetes from caves in the remediation of complex polymers, heavy metal and organic pollutant-contaminated Soils.

Actinomycetes play relevant ecological roles in environments, including the recycling of substances and degradation of pollutants. Actinobacteria can remove organic and inorganic pollutants (i.e., pesticides and heavy metals) [94]. Beyond bioactive molecules, actinomycetes produce enzyme inhibitors, immunosuppressors, phytotoxins, biopesticides, biosurfactants, probiotics, and enzymes involved in the degradation of complex polymers [95]. These capabilities are promoted by the oligotrophic properties of caves that stimulate inimitable strategies of the indigenous microbiome to remove pollutant compounds [24,96]. Augmentation, stimulation, cell immobilisation, and the production of biosurfactants were exploited during the last decades to enhance the capabilities of actinobacteria in bioremediation [97]. Figure 4 summarises the possible uses of actinomycetes from caves in the remediation of complex polymers, heavy metals, and organic pollutant-contaminated soils, which are described in the following paragraphs.

### 4.1. Heavy Metals

Within caves, heavy metal stress is a critical environmental factor determining the structure and function of microbial communities [98]. Oliveira and Pampulha reported a marked decrease of the different microbial groups for contaminated soil samples compared to uncontaminated samples [99]. The heavy metal contamination also negatively affects microbial metabolic abilities. Ellis and collaborators found that metal contamination did not significantly affect the total genetic diversity present but affected physiological conditions of culturable microflora. The number of bacteria capable of responding to laboratory culture and their taxonomic distribution were significantly altered [100].

Actinomycetes, together with proteobacteria, are the representative constituents of physiologically active fractions in sites contaminated with heavy metals [101]. Among cave actinomycetes tolerant to heavy metal stress, *Streptomyces* shows a remarkable capacity to tolerate a wide range and different concentrations of heavy metals. The recent work of Hamedi and collaborators reported that about 26% of actinomycetes’ strains isolated in soil and water of different sections of Hampoeil cave (Iran) could tolerate Pb (5 mM), Ni (15 mM), Cd (2.5 mM), Cu (3 mM), and Zn (35 mM) [24]. In this study, the authors found that *Nocardia* sp. UTMC 3191 and *Streptomyces* sp. UTMC 3261 strains were able to tolerate all five different heavy metals, while *Micromonospora soli* UTMC 3168, *Streptomyces* sp. UTMC 3178 and *Streptomyces pratensis* UTMC 3254 tolerated only three of them (Zn, Cu, Ni). Among them, some *Actinobacteria* reached a maximum tolerance to the heavy metals tested relative to other strains. For instance, *Micromonospora aurantiaca* UTMC 3161, *Nocardia* sp. UTMC 3191 and *Streptomyces pratensis* UTMC 3254 showed the maximum resistance to Zn (70 mM) in comparison to 35 mM for other isolates. Likewise, *Micromonospora* sp. UTMC 3162, *Streptomyces* sp. UTMC 3164, *Micromonospora soli* UTMC 3168, *Rhodococcus* sp. UTMC 3171, *Streptomyces* sp. UTMC 3178, and *Nocardia* sp. UTMC 3191 had the highest Ni tolerance (30 mM) compared to 35 mM for other strains. Possible evidence that can help in speculating the use of metal-tolerant actinomycetes as bioremediation agents exists in the literature on mines. Even if species’ composition between cave and mine environments is different, the heavy metal tolerance is similar.

Evidence recorded from mines has demonstrated that *Streptomyces* is the most efficient genus of actinomycetes that can tolerate heavy metals. Hurtado and collaborators investigated 24 strains of actinomycetes, isolated from arsenopyrite minerals from different mining areas of Peru, to evaluate their potential in bioleaching processes of arsenopyrite [102]. Among the isolated strains, *Streptomyces* sp. E1 and *Streptomyces variabilis* AB5 were able to develop in arsenopyrite. In particular, the leached solutions of *Streptomyces* sp. E1 and *S. variabilis* AB5 showed an arsenic (As) extraction present in arsenopyrite of 19.1% and 15.5%, respectively. The ability of *Streptomyces* to tolerate heavy metal stress was also confirmed for thorium (Th) and uranium (U), two actinide elements that have become the centre of broad interest in the recovery of nuclear fuel elements and the removal of nuclear wastes. Nakajima and Tsuruta examined the competitive biosorption of thorium and uranium by actinomycetes. They observed that *Streptomyces levoris* had the highest ability to sorb the two metals in metal-single and metal-mixed solutions from aqueous systems [103]. Undabarrena and collaborators analysed genetic determinants involved in heavy metal tolerance in the *Streptomyces* sp. H-KF8 strain by genome mining and studying 49 predicted genes mainly related to arsenate, copper, and mercury tolerance. In this study, the authors found that arsenic tolerance involved three *arsC* genes encoding arsenate reductases, two *arsA* genes encoding arsenical pump driving ATPases, five *arsR* genes encoding arsenical transcriptional regulators, and the arsenical resistance protein encoding gene *acr3*. Among copper resistance genes, *copA* and *mco* genes encoding multicopper oxidases, *copD* gene encoding a copper resistance protein, two *ycnJ* genes encoding for copper transport proteins, and two *csoR* genes for the copper-sensing transcriptional regulator are determinant factors [104]. These findings pave the way to using cave actinomycetes for heavy metal-contaminated soil remediation.

### 4.2. Organic Pollutants

Intensive agriculture techniques and production, fertilisation, and industrial wastewaters (e.g., crude oil refineries, coal gasification plants) often produce components of environmental pollution (e.g., carbamate, phenols, organochlorine, and organophosphorus) [105]. Despite the lack of records dealing with the organic pollutant-degradative strains from caves, actinomycetes are considered the ideal bioremediation agents because of their metabolic diversity and peculiar growth and survival [106]. Among pesticide-degrading actinobacteria, *Arthrobacter*, *Rhodococcus*, *Streptomyces*, *Frankia*, *Janibacter*, *Kocuria*, *Mycobacterium*, *Nocardia*, and *Pseudonocardia* are the most representative. In particular, the genus, *Arthrobacter*, has been recognised as a degrader of different xenobiotics since members of this group possess various catabolic pathways to detoxify these compounds [107,108,109]. For instance, an Arthrobacter strain (*A. chlorophenolicus* A6) is a good candidate for 4-chlorophenol-degradation activity. Bjerketorp et al. investigated the viability of formulating and stabilising a product based on dried *A. chlonophenolius* A6 in soils contaminated with 4-CP, that may be useful for the development of less cost and less technically challenging remediation techniques. This formulation, based on micronised vermiculite, showed survival rates of about 60% and remained stable in storage for at least 3 months at 4 °C. Furthermore, in controlled-environment soil microcosm, the stabilised cells degraded 4-CP as efficiently as freshly grown cells. In fact, it reduced the initial concentration of 130 µg 4-CP g^−1^ dry soil to a final concentration of 20 µg 4-CP g^−1^ dry soil after 13 days [110].

Baoune et al. used *Streptomyces* sp. Hlh1 strain to test its capacity to remediate petroleum-contaminated soil. They discovered that this strain could grow and remove total petroleum hydrocarbons (TPH), including n-Alkanes (C6-C35) and 14 priority aromatic hydrocarbons (PAHs). The experiments were carried out in both sterilised and non-sterile soils. The strain removed up to 40% and 55% of TPH under sterile and non-sterile conditions, respectively [111]. As previously reported, actinobacteria can recover different environmental matrices contaminated by organophosphorus pesticides. Briceño et al. examined the *Streptomyces* spp. consortium ability to remove chlorpyrifos and diazinon from liquid, soil, and biobed mixtures. From liquid, a removal rate of 0.036 h ^-1^ and 0.015 h ^-1^ and a half-life of 19 h and 46 h were recorded for chlorpyrifos and diazinon, respectively. For soil and biobed mixtures, limited chlorpyrifos removal was achieved (6–14 %), whereas diazinon showed a removal rate of 0.024 day ^−1^ and 0.060 day ^−1^ and a half-life of 29 and 11 days [112]. Among actinobacteria, rhodococci have practical industrial and ecological applications due to their various metabolic activities. Members of the genus, *Rhodococcus*, are ubiquitous in fertile soil and can also be present in polluted environments where they play an essential role in degrading different pollutants [113]. Rhodococci are promising candidates for bioremediation due to their resistance to starvation in soil, while carbon sources that are more simply assimilable might not negatively affect the contaminants’ breakdown [114]. Different *Streptomyces* strains can grow on and degrade several classes of pesticides [115].

## 5. Limitations and Advantages in the Use of Actinomycetes

The use of actinomycetes-based products and, more generally, bacterial-based products for soil environments applications present both advantages and disadvantages. Compared to agrochemicals and other physical and chemical treatments used for agriculture or remediation/restoration, actinomycetes-based products are sustainable. Actinomycetes-based products are entirely eco-friendly and not harmful for organisms [106]. Compared to other bacterial-based products, actinomycetes have great versatility and particular cell properties [2]. These characteristics pave the road to more possibilities to find the characteristics of the ideal strain for industrialisation and the production of formulations. Even if these advantages are essential, some limitations can occur in microbial formulations in soil environments. Unlike synthetic chemicals, the optimisation, formulation, and preparation of inoculants can be challenging and must follow several rigorous validation steps and the industrial scaling-up of bioreactions [55]. The effectiveness of bacterial inoculants as biostimulants and biocontrol agents in soil environments presents high variability as bacteria are susceptible to abiotic and biotic factors. The rate of effectiveness is not comparable to that of chemical fertilisers. However, these limitations are less heavy for actinomycete-based products. Since actinomycetes are spore-forming bacteria, most actinomycetes can survive, adapt, and be effective under diverse environmental conditions [57]. Because of their many functions, summarised in Table 3, actinomycetes are a significant source for developing a wide range of technological applications. 

However, there are some drawbacks. A good portion of actinomycetes from subterranean environments is not cultivable, it includes still unknown species, and there are still no reliable and efficient cultivation protocols [117]. Another crucial aspect of cave actinomycetes is connected to human health because of their potential pathogenicity. Several actinobacterial species, mostly belonging to the genera *Gordonia*, *Mycobacterium*, *Nocardia*, *Rhodococcus*, and *Streptomyces*, are known to cause skin, lung, and brain infections, and bronchopulmonary diseases to humans [118]. Actinomycetes may also represent a potential risk for natural and cultural heritage beneath the surface. Several studies have demonstrated that their metabolism’s organic and inorganic products negatively affect cave paintings and secondary deposits, such as stalactites and stalagmites [116,119]. Pinzari and collaborators also detected some actinobacteria species as the cause of the parchment discolouration [120]; this phenomenon consists of the occurrence of purple spots on the parchment, and the proliferation of actinobacteria could also provide favourable conditions for the development of proteolytic and mitosporic fungi that contribute to the deterioration process [121]. However, this limit can be overcome, as it is mainly understood that the study of cave actinomycetes is exceptionally young and has been growing in recent years, at the same rate with the new technologies. Therefore, if added to the great potential of actinomycetes, these criticalities only intensify the motivation to deepen the study of this fascinating taxon, and consequently, to invest resources in this promising and multifaceted field.

## 6. Conclusions and Future Perspectives

Over the past decades, subterranean ecosystems have raised interest because of the dwelling microbiome that these environments conceal in the dark. In such unfavourable environments, studies on autecological and synecological responses have revealed new insights for developing advanced biotechnological applications. From the evidence summarised in this review, it is evident that caves represent a crucial window to investigate actinomycetes and their pharmacologically, clinically, and agriculturally relevant bioactive compounds. Most of the published studies address antibiotic potentialities towards human pathogens and the production of bioactive compounds for human health. However, these bacteria can be a source of compounds beneficial for soil environments applications, including bioremediation and agriculture. Due to their metabolic versatility and resistance under diverse stressful conditions, actinomycetes from caves have a significant potential to be used as biostimulant, biocontrol, and bioremediation agents in soil environments. The survey on the literature also showed that the *Streptomyces* genus seems to be the most promising target of study due to ubiquity, survival capabilities, and metabolic versatility. Despite these potentialities, this review highlighted the continued need to retain poor information concerning distribution, population dynamics, biogeochemical processes, and metabolisms of cave actinomycetes. The limited information obtained on soil is not scientifically sufficient enough to support cave actinomycetes in biotechnological applications. In our opinion, research on cave actinomycetes needs to be encouraged, especially concerning environmental soil applications to improve soil fertility and health, and to antagonise phytopathogens.

## Figures and Tables

**Table 1:** Summary of the actinomycetes isolated from caves.

Strains	Cave	Location	Activity	Reference
*Streptomyces* spp. 52%; *Actinoplanes* spp. 13%; *Nocardioides* 8.7%; *Micromonospora* 8.7%; *Agromyces* 8.7%; *Oerskovia* 4.4%; *Rhodococcus* 4.4%	Shuanghe Cave	Asia	Antimicrobial	[23]
*Streptomyces* (54%); *Micromonospora* (18%); *Micrococcus* (5%); *Kocuria* (4%); *Corynebacterium* (5%)	Hampoeil cave	Iran	Heavy metal tolerance	[24]
*Knoellia sinensis* gen. nov. sp. nov.; *Knoellia subterranea* sp. nov.	Reed Flute Cave	China		[25]
*Streptomyces*; *Agromyces*; *Arthrobacter*; *Rhodococcus*	Grotta dei Cervi	Italy	Halotolerance Mineralisation	[26]
*Streptomyces* (79.3%); *Nocardia* (<10%)	Helmcken Falls cave	Canada	Antimicrobial	[27]
*Prauserella* spp.	Cave of Crystals	Mexico		[28]
*Streptomyces* (main) *Nocardia*; *Rhodococcus*; *Nocardioides*; *Amycolatopsis*; *Saccharothrix*; *Brevibacterium*; *Microbacterium*; coccoid actinomycetes (family *Micrococcaceae*)	Altamira and Tito Bustillo	Spain		[29]
*Streptomyces*; *Micromonospora*; *Microbacterium*; *Kocuria*; *Micrococcus*; *Nocardiopsis*; *Brevibacterium*	Pukzing cave	India	Antimicrobial	[30]
*Streptomyces* (mainly) *Actinocorallia*; *Actinomadura*; *Agromyces*; *Alloactinosynnema*; *Amycolatopsis*; *Beutenbergia*; *Cellulosimicrobium*; *Gordonia*; *Isoptericola*; *Jiangella*; *Knoellia*; *Kocuria*; *Krasilnikoviella*; *Kribbella*; *Microbacterium*; *Micromonospora*; *Mumia*; *Mycobacterium*; *Nocardia*; *Nocardioides*; *Nocardiopsis*; *Nonomuraea*; *Oerskovia*; *Pseudokineococcus*; *Pseudonocardia*; *Rhodococcus*; *Saccharothrix*; *Streptosporangium*; *Tsukamurella*	Sigangli Cave	China		[31]
*Microbispora thailandensis* sp. nov.	Tropical limestone caves	Thailand	Antimicrobial	[32]
*Nonomuraea antri* sp. nov.	Tropical limestone caves	Thailand		[33]

**Table 2:** Summary of the actinomycetes characterised in caves microbiota.

Actinobacteria Described	Abundance of Actinomycetes in Bacterial Community	Cave	Location	References
*Mycobacterium* 29.9%; *Nocardioides* 21.9%; *Streptomyces* spp. 15.1%	42–48%	Shuanghe Cave	Asia	[23]
*Streptomyces* within the dominant order of *Streptomycetales*	93%	Pukzing cave	India	[30]
*Arthrobacter*; *Acidimicrobidae*; *Actinosynnemataceae*; *Brevibacterium*; *Frankia*; *Kocuria*; *Microbacteriaceae*; *Micrococcaceae*; *Nocardiaceae*; *Nocardioidaceae*; *Pseudonocardiaceae*; *Streptomycetaceae*; *Saccharothrix*; *Rhodococcus*	>25%	Pajsarjeva jama	Slovenia	[20]
*Actinomycetales* order dominance	-	Different caves	Spain, Portugal, USA, Canada	[22]
*Mycobacterium*; *Corynebacterium*; *Rubrobacter*; *Actinoplanes*; *Saccharothrix*; *Pseudonocardia*	Up to 65%	Five different caves	India	[34]
-	14–34%	Bellavista and Royal Palm Caves	Ecuador	[35]
-	2–34%	Pertosa-Auletta Cave	Italy	[36]

**Table 3:** Summary of the functions of culturable genera actinomycetes isolated from caves.

Genera	Functions	References
*Streptomyces* spp.	Antibacterial, antioxidant, and anticancer bioactive molecules. Effective use of metabolites against phytopathogens. Biotic and abiotic stress tolerance. Plant growth-promoting activities. Soil bioconsolidation and bioremediation.	[18,24,45,53,55,57,68,97,103,116]
*Micromonospora* spp.	Pollutants’ degradation and soil detoxification.	[24,96,105]
*Nocardia* spp.		[24,105,116]
*Nocardiopsis* spp.		[68]
*Arthrobacter* spp.		[105]
*Rhodococcus* spp.		[105,116]
*Frankia* spp.		[105]
*Kocuria* spp.		[105]
*Janibacter* spp.		[105]
*Pseudonocardia* spp.		[105]
